# *N*-Cyclo­hexyl-*N*-ethyl­benzene­sulfonamide

**DOI:** 10.1107/S1600536809044006

**Published:** 2009-10-28

**Authors:** Islam Ullah Khan, Zeeshan Haider, Muhammad Zia-ur-Rehman, Muhammad Nadeem Arshad, Muhammad Shafiq

**Affiliations:** a Department of Chemistry, Government College University, Lahore 54000, Pakistan; b Applied Chemistry Research Centre, PCSIR Laboratories Complex, Ferozpure Road, Lahore 54600, Pakistan.

## Abstract

The title compound, C_14_H_21_NO_2_S, synthesized by *N*-methyl­ation of cyclo­hexyl­amine sulfonamide with ethyl iodide is of inter­est as a precursor to biologically active sulfur-containing heterocyclic compounds. There are two independent mol­ecules in the asymmetric unit. The dihedral angles between the mean planes of the phenyl ring and the cyclo­hexyl ring are 40.29 (11) and 37.91 (13)° in the two mol­ecules.

## Related literature

For the synthesis of related mol­ecules, see: Arshad *et al.* (2009 ▶); Zia-ur-Rehman *et al.* (2009 ▶). For the biological activity of sulfonamides, see: Berredjem *et al.* (2000 ▶); Lee & Lee (2002 ▶); Soledade *et al.* (2006 ▶); Xiao & Timberlake (2000 ▶).
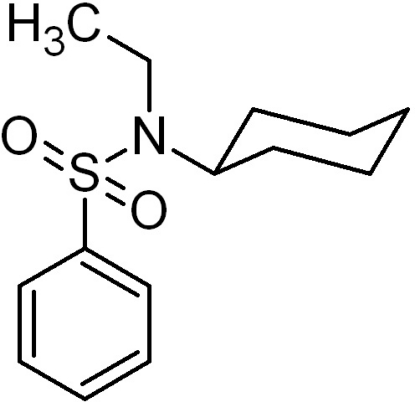


## Experimental

### 

#### Crystal data

C_14_H_21_NO_2_S
*M* *_r_* = 267.38
Monoclinic, 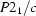

*a* = 18.195 (1) Å
*b* = 12.9799 (7) Å
*c* = 12.7327 (6) Å
β = 108.587 (3)°
*V* = 2850.2 (3) Å^3^
*Z* = 8
Mo *K*α radiation
μ = 0.22 mm^−1^
*T* = 296 K
0.42 × 0.11 × 0.08 mm

#### Data collection

Bruker APEXII CCD area-detector diffractometer
Absorption correction: none
32427 measured reflections
7061 independent reflections
3979 reflections with *I* > 2σ(*I*)
*R*_int_ = 0.056

#### Refinement

*R*[*F* ^2^ > 2σ(*F* ^2^)] = 0.052
*wR*(*F* ^2^) = 0.148
*S* = 0.99
7061 reflections
327 parameters
H-atom parameters constrained
Δρ_max_ = 0.30 e Å^−3^
Δρ_min_ = −0.24 e Å^−3^

### 

Data collection: *APEX2* (Bruker, 2007 ▶); cell refinement: *SAINT* (Bruker, 2007 ▶); data reduction: *SAINT*; program(s) used to solve structure: *SHELXS97* (Sheldrick, 2008 ▶); program(s) used to refine structure: *SHELXL97* (Sheldrick, 2008 ▶); molecular graphics: *PLATON* (Spek, 2009 ▶) and *Mercury* (Macrae *et al.*, 2006 ▶); software used to prepare material for publication: *SHELXTL* (Sheldrick, 2008 ▶) and local programs.

## Supplementary Material

Crystal structure: contains datablocks I, global. DOI: 10.1107/S1600536809044006/bt5111sup1.cif

Structure factors: contains datablocks I. DOI: 10.1107/S1600536809044006/bt5111Isup2.hkl

Additional supplementary materials:  crystallographic information; 3D view; checkCIF report

## Figures and Tables

**Fig. 1.**
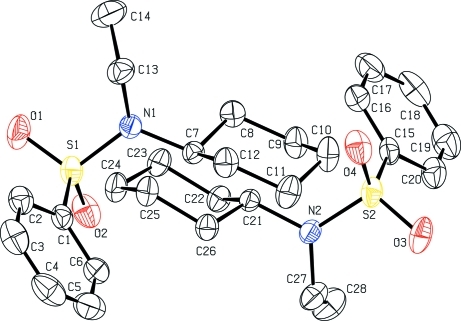
The molecular structure of the title compound, with displacement ellipsoids at the 50% probability level. H atoms have been omitted for clarity.

## supplementary crystallographic information

### Comment

Sulfonamide is an important functionality found in a number of synthetic as well as natural compounds possessing versatile type of biological activities *e.g.*, herbicidal, anti-malarial, anti-convulsant and anti-hypertensive (Soledade *et al.*, 2006; Xiao & Timberlake, 2000; Berredjem *et al.*, 2000; Lee & Lee, 2002) activities.

As a part of our ongoing research program regarding the synthesis of sulfur containing heterocyclic compounds (Arshad *et al.*, 2009; Zia-ur-Rehman *et al.* 2009), we, herein report the crystal structure of the title compound (Scheme and figure1). Bond lengths and bond angles are within the normal ranges. No significant hydrogen bond interactions are observed in the title molecule. The dihedral angles between the mean planes of the phenyl ring and   the cyclohexyl ring  are 40.29 (11)° and 37.91 (13)°, respectively, for the two molecules in the asymmetric unit.

### Experimental

A mixture of *N*-cyclohexylbenzene sulfonamide1(g, 0.43 mmol), sodium hydride (0.21 g; 0.88 mmoles) and *N*, *N*-dimethylformamide (10.0 ml) was stirred at room temperature for half an hour followed by addition of ethyl iodide (0.134 g; 0.86 mmoles). Stirring was continued further for a period of three hours and the contents were poured over crushed ice. The precipitated product was isolated, washed and crystallized from a methanol-water mixture (50: 50).

### Refinement

All hydrogen atoms were identified in the difference map. However, they were fixed in ideal positions and treated as riding on their parent atoms. The following distances were used: C_methyl_—H = 0.98Å;  C_aromatic_—H = 0.95Å. U(H) was set to 1.2U_eq_(C) or 1.5U_eq_(C_methyl_).

### Crystal data

**Table:**

C_14_H_21_NO_2_S	*F*(000) = 1152
*M**_r_* = 267.38	*D*_x_ = 1.246 Mg m^−^^3^
Monoclinic, *P*2_1_/*c*	Mo *K*α radiation, λ = 0.71073 Å
Hall symbol: -P 2ybc	Cell parameters from 3394 reflections
*a* = 18.195 (1) Å	θ = 2.3–21.8°
*b* = 12.9799 (7) Å	µ = 0.22 mm^−^^1^
*c* = 12.7327 (6) Å	*T* = 296 K
β = 108.587 (3)°	Needles, colourless
*V* = 2850.2 (3) Å^3^	0.42 × 0.11 × 0.08 mm
*Z* = 8	

### Data collection

**Table:**

Bruker APEXII CCD area-detector diffractometer	3979 reflections with *I* > 2σ(*I*)
Radiation source: fine-focus sealed tube	*R*_int_ = 0.056
graphite	θ_max_ = 28.3°, θ_min_ = 1.2°
φ and ω scans	*h* = −24→23
32427 measured reflections	*k* = −17→16
7061 independent reflections	*l* = −16→17

### Refinement

**Table:**

Refinement on *F*^2^	Primary atom site location: structure-invariant direct methods
Least-squares matrix: full	Secondary atom site location: difference Fourier map
*R*[*F*^2^ > 2σ(*F*^2^)] = 0.052	Hydrogen site location: inferred from neighbouring sites
*wR*(*F*^2^) = 0.148	H-atom parameters constrained
*S* = 0.99	*w* = 1/[σ^2^(*F*_o_^2^) + (0.0572*P*)^2^ + 0.5289*P*] where *P* = (*F*_o_^2^ + 2*F*_c_^2^)/3
7061 reflections	(Δ/σ)_max_ < 0.001
327 parameters	Δρ_max_ = 0.30 e Å^−^^3^
0 restraints	Δρ_min_ = −0.24 e Å^−^^3^

### Special details

**Table:**

Geometry. All e.s.d.'s (except the e.s.d. in the dihedral angle between two l.s. planes) are estimated using the full covariance matrix. The cell e.s.d.'s are taken into account individually in the estimation of e.s.d.'s in distances, angles and torsion angles; correlations between e.s.d.'s in cell parameters are only used when they are defined by crystal symmetry. An approximate (isotropic) treatment of cell e.s.d.'s is used for estimating e.s.d.'s involving l.s. planes.
Refinement. Refinement of *F*^2^ against ALL reflections. The weighted *R*-factor *wR* and goodness of fit *S* are based on *F*^2^, conventional *R*-factors *R* are based on *F*, with *F* set to zero for negative *F*^2^. The threshold expression of *F*^2^ > σ(*F*^2^) is used only for calculating *R*-factors(gt) *etc*. and is not relevant to the choice of reflections for refinement. *R*-factors based on *F*^2^ are statistically about twice as large as those based on *F*, and *R*- factors based on ALL data will be even larger.

### Fractional atomic coordinates and isotropic or equivalent isotropic displacement parameters (Å^2^)

**Table:**

	*x*	*y*	*z*	*U*_iso_*/*U*_eq_	
S1	0.36993 (4)	0.68047 (5)	0.03599 (5)	0.0533 (2)	
O1	0.39686 (11)	0.64718 (16)	−0.05209 (14)	0.0755 (6)	
O2	0.37154 (11)	0.78735 (14)	0.06200 (17)	0.0710 (6)	
N1	0.28139 (11)	0.64066 (15)	0.00768 (15)	0.0464 (5)	
C1	0.42614 (14)	0.61647 (19)	0.1568 (2)	0.0473 (6)	
C2	0.46699 (15)	0.5285 (2)	0.1493 (2)	0.0574 (7)	
H2	0.4652	0.5029	0.0803	0.069*	
C3	0.51032 (18)	0.4790 (2)	0.2447 (3)	0.0751 (9)	
H3	0.5381	0.4200	0.2401	0.090*	
C4	0.5126 (2)	0.5163 (3)	0.3462 (3)	0.0848 (11)	
H4	0.5420	0.4826	0.4103	0.102*	
C5	0.47221 (19)	0.6025 (3)	0.3537 (2)	0.0793 (10)	
H5	0.4737	0.6270	0.4230	0.095*	
C6	0.42914 (16)	0.6539 (2)	0.2599 (2)	0.0614 (7)	
H6	0.4022	0.7133	0.2657	0.074*	
C7	0.23333 (13)	0.68049 (18)	0.07358 (17)	0.0409 (6)	
H7	0.2614	0.7389	0.1167	0.049*	
C8	0.15687 (14)	0.7218 (2)	−0.00157 (19)	0.0529 (7)	
H8A	0.1276	0.6663	−0.0470	0.063*	
H8B	0.1666	0.7735	−0.0505	0.063*	
C9	0.10942 (16)	0.7692 (2)	0.0651 (2)	0.0637 (8)	
H9A	0.1357	0.8302	0.1029	0.076*	
H9B	0.0593	0.7902	0.0151	0.076*	
C10	0.09773 (17)	0.6946 (2)	0.1492 (2)	0.0725 (9)	
H10A	0.0714	0.7294	0.1944	0.087*	
H10B	0.0650	0.6383	0.1110	0.087*	
C11	0.17359 (17)	0.6522 (2)	0.2226 (2)	0.0701 (8)	
H11A	0.1637	0.6011	0.2719	0.084*	
H11B	0.2036	0.7074	0.2677	0.084*	
C12	0.22025 (15)	0.6035 (2)	0.1559 (2)	0.0572 (7)	
H12A	0.1928	0.5439	0.1164	0.069*	
H12B	0.2699	0.5805	0.2056	0.069*	
C13	0.26176 (16)	0.5405 (2)	−0.0484 (2)	0.0591 (7)	
H13A	0.2257	0.5049	−0.0191	0.071*	
H13B	0.3084	0.4990	−0.0317	0.071*	
C14	0.2265 (2)	0.5491 (3)	−0.1719 (2)	0.0908 (11)	
H14A	0.1821	0.5936	−0.1892	0.136*	
H14B	0.2109	0.4821	−0.2028	0.136*	
H14C	0.2640	0.5773	−0.2026	0.136*	
S2	0.14463 (4)	0.28954 (5)	0.47894 (6)	0.0536 (2)	
O3	0.12606 (12)	0.31641 (16)	0.57629 (15)	0.0769 (6)	
O4	0.13794 (11)	0.18488 (13)	0.44305 (17)	0.0707 (6)	
N2	0.23252 (11)	0.32654 (14)	0.49770 (15)	0.0455 (5)	
C15	0.08332 (14)	0.36211 (19)	0.3684 (2)	0.0501 (6)	
C16	0.07673 (16)	0.3370 (2)	0.2607 (2)	0.0635 (8)	
H16	0.1030	0.2803	0.2456	0.076*	
C17	0.03073 (18)	0.3967 (3)	0.1753 (3)	0.0799 (9)	
H17	0.0267	0.3809	0.1024	0.096*	
C18	−0.00889 (18)	0.4787 (3)	0.1970 (3)	0.0850 (10)	
H18	−0.0405	0.5181	0.1391	0.102*	
C19	−0.00210 (19)	0.5031 (3)	0.3045 (3)	0.0829 (10)	
H19	−0.0291	0.5592	0.3192	0.099*	
C20	0.04401 (17)	0.4456 (2)	0.3902 (3)	0.0670 (8)	
H20	0.0488	0.4628	0.4630	0.080*	
C21	0.27320 (13)	0.29122 (17)	0.42041 (17)	0.0398 (6)	
H21	0.2384	0.2427	0.3690	0.048*	
C22	0.29029 (15)	0.37719 (19)	0.35061 (19)	0.0498 (6)	
H22A	0.2425	0.4123	0.3108	0.060*	
H22B	0.3250	0.4269	0.3985	0.060*	
C23	0.32733 (15)	0.3341 (2)	0.26870 (19)	0.0547 (7)	
H23A	0.2902	0.2905	0.2157	0.066*	
H23B	0.3408	0.3904	0.2282	0.066*	
C24	0.39939 (15)	0.2723 (2)	0.3260 (2)	0.0601 (7)	
H24A	0.4185	0.2408	0.2706	0.072*	
H24B	0.4393	0.3181	0.3705	0.072*	
C25	0.38391 (16)	0.1893 (2)	0.3992 (2)	0.0574 (7)	
H25A	0.4324	0.1561	0.4398	0.069*	
H25B	0.3501	0.1375	0.3533	0.069*	
C26	0.34638 (14)	0.23229 (19)	0.48063 (19)	0.0489 (6)	
H26A	0.3338	0.1762	0.5223	0.059*	
H26B	0.3826	0.2777	0.5325	0.059*	
C27	0.26849 (18)	0.4076 (2)	0.5788 (2)	0.0659 (8)	
H27A	0.3243	0.4032	0.5960	0.079*	
H27B	0.2567	0.3936	0.6466	0.079*	
C28	0.2440 (2)	0.5137 (2)	0.5434 (3)	0.0844 (10)	
H28A	0.2526	0.5275	0.4742	0.127*	
H28B	0.2736	0.5613	0.5985	0.127*	
H28C	0.1899	0.5215	0.5346	0.127*	

### Atomic displacement parameters (Å^2^)

**Table:**

	*U*^11^	*U*^22^	*U*^33^	*U*^12^	*U*^13^	*U*^23^
S1	0.0519 (4)	0.0526 (4)	0.0639 (4)	0.0001 (3)	0.0304 (3)	0.0080 (3)
O1	0.0719 (14)	0.1040 (16)	0.0669 (12)	0.0096 (12)	0.0452 (10)	0.0145 (11)
O2	0.0605 (13)	0.0438 (11)	0.1111 (15)	−0.0072 (10)	0.0307 (11)	0.0086 (10)
N1	0.0459 (13)	0.0466 (13)	0.0501 (11)	−0.0015 (10)	0.0200 (9)	−0.0048 (9)
C1	0.0412 (15)	0.0473 (16)	0.0577 (15)	−0.0071 (13)	0.0218 (12)	−0.0012 (12)
C2	0.0539 (18)	0.0514 (17)	0.0708 (18)	−0.0053 (15)	0.0256 (15)	0.0023 (14)
C3	0.059 (2)	0.062 (2)	0.106 (3)	0.0033 (16)	0.0268 (18)	0.0164 (19)
C4	0.064 (2)	0.096 (3)	0.082 (2)	−0.021 (2)	0.0061 (18)	0.029 (2)
C5	0.072 (2)	0.099 (3)	0.0605 (19)	−0.024 (2)	0.0135 (17)	0.0000 (19)
C6	0.0545 (18)	0.0647 (19)	0.0678 (18)	−0.0144 (15)	0.0236 (15)	−0.0111 (15)
C7	0.0436 (14)	0.0394 (14)	0.0427 (12)	−0.0033 (11)	0.0179 (11)	−0.0017 (10)
C8	0.0531 (16)	0.0558 (17)	0.0501 (13)	0.0040 (14)	0.0168 (12)	0.0076 (12)
C9	0.0546 (18)	0.070 (2)	0.0715 (17)	0.0138 (15)	0.0270 (14)	0.0101 (15)
C10	0.061 (2)	0.086 (2)	0.084 (2)	0.0137 (17)	0.0427 (16)	0.0132 (17)
C11	0.081 (2)	0.079 (2)	0.0640 (17)	0.0060 (18)	0.0433 (16)	0.0147 (15)
C12	0.0603 (18)	0.0580 (17)	0.0589 (15)	0.0040 (15)	0.0268 (13)	0.0149 (13)
C13	0.0666 (19)	0.0513 (17)	0.0613 (16)	0.0017 (14)	0.0228 (14)	−0.0094 (13)
C14	0.109 (3)	0.096 (3)	0.0632 (19)	0.005 (2)	0.0218 (18)	−0.0250 (18)
S2	0.0558 (4)	0.0487 (4)	0.0696 (4)	−0.0011 (3)	0.0386 (3)	0.0054 (3)
O3	0.0871 (15)	0.0920 (15)	0.0755 (12)	0.0103 (12)	0.0596 (11)	0.0152 (11)
O4	0.0642 (13)	0.0413 (11)	0.1158 (15)	−0.0094 (10)	0.0415 (11)	0.0017 (10)
N2	0.0521 (13)	0.0427 (12)	0.0492 (11)	−0.0066 (10)	0.0267 (9)	−0.0077 (9)
C15	0.0408 (15)	0.0497 (16)	0.0676 (16)	−0.0033 (13)	0.0283 (13)	−0.0019 (13)
C16	0.0528 (18)	0.069 (2)	0.0711 (18)	−0.0079 (16)	0.0239 (15)	−0.0120 (16)
C17	0.051 (2)	0.109 (3)	0.071 (2)	−0.010 (2)	0.0067 (16)	0.004 (2)
C18	0.047 (2)	0.083 (3)	0.112 (3)	−0.0060 (19)	0.0077 (19)	0.027 (2)
C19	0.061 (2)	0.067 (2)	0.121 (3)	0.0084 (18)	0.029 (2)	0.000 (2)
C20	0.0582 (19)	0.066 (2)	0.083 (2)	0.0117 (16)	0.0302 (16)	−0.0007 (17)
C21	0.0427 (14)	0.0390 (13)	0.0422 (12)	−0.0044 (11)	0.0197 (10)	−0.0044 (10)
C22	0.0575 (17)	0.0498 (15)	0.0476 (13)	0.0004 (13)	0.0245 (12)	0.0043 (12)
C23	0.0635 (18)	0.0614 (18)	0.0476 (13)	0.0016 (15)	0.0297 (13)	0.0063 (12)
C24	0.0572 (18)	0.0721 (19)	0.0619 (15)	0.0006 (15)	0.0344 (13)	0.0032 (14)
C25	0.0535 (17)	0.0617 (18)	0.0647 (15)	0.0106 (14)	0.0298 (13)	0.0053 (14)
C26	0.0500 (16)	0.0511 (16)	0.0496 (13)	0.0011 (13)	0.0217 (12)	0.0058 (12)
C27	0.085 (2)	0.0609 (19)	0.0617 (16)	−0.0021 (17)	0.0371 (15)	−0.0122 (15)
C28	0.115 (3)	0.060 (2)	0.085 (2)	0.0096 (19)	0.041 (2)	−0.0057 (17)

### Geometric parameters (Å, °)

**Table:**

S1—O2	1.4244 (19)	S2—O4	1.4261 (18)
S1—O1	1.4266 (18)	S2—O3	1.4280 (17)
S1—N1	1.619 (2)	S2—N2	1.613 (2)
S1—C1	1.761 (3)	S2—C15	1.764 (3)
N1—C13	1.472 (3)	N2—C27	1.473 (3)
N1—C7	1.485 (3)	N2—C21	1.480 (3)
C1—C2	1.382 (3)	C15—C20	1.375 (4)
C1—C6	1.384 (3)	C15—C16	1.377 (3)
C2—C3	1.378 (4)	C16—C17	1.379 (4)
C2—H2	0.9300	C16—H16	0.9300
C3—C4	1.368 (4)	C17—C18	1.363 (5)
C3—H3	0.9300	C17—H17	0.9300
C4—C5	1.359 (5)	C18—C19	1.371 (5)
C4—H4	0.9300	C18—H18	0.9300
C5—C6	1.374 (4)	C19—C20	1.367 (4)
C5—H5	0.9300	C19—H19	0.9300
C6—H6	0.9300	C20—H20	0.9300
C7—C8	1.513 (3)	C21—C26	1.515 (3)
C7—C12	1.520 (3)	C21—C22	1.519 (3)
C7—H7	0.9800	C21—H21	0.9800
C8—C9	1.520 (3)	C22—C23	1.517 (3)
C8—H8A	0.9700	C22—H22A	0.9700
C8—H8B	0.9700	C22—H22B	0.9700
C9—C10	1.508 (4)	C23—C24	1.512 (4)
C9—H9A	0.9700	C23—H23A	0.9700
C9—H9B	0.9700	C23—H23B	0.9700
C10—C11	1.503 (4)	C24—C25	1.508 (3)
C10—H10A	0.9700	C24—H24A	0.9700
C10—H10B	0.9700	C24—H24B	0.9700
C11—C12	1.517 (3)	C25—C26	1.519 (3)
C11—H11A	0.9700	C25—H25A	0.9700
C11—H11B	0.9700	C25—H25B	0.9700
C12—H12A	0.9700	C26—H26A	0.9700
C12—H12B	0.9700	C26—H26B	0.9700
C13—C14	1.501 (4)	C27—C28	1.474 (4)
C13—H13A	0.9700	C27—H27A	0.9700
C13—H13B	0.9700	C27—H27B	0.9700
C14—H14A	0.9600	C28—H28A	0.9600
C14—H14B	0.9600	C28—H28B	0.9600
C14—H14C	0.9600	C28—H28C	0.9600
			
O2—S1—O1	119.41 (12)	O4—S2—O3	119.41 (12)
O2—S1—N1	107.93 (11)	O4—S2—N2	108.09 (11)
O1—S1—N1	107.15 (11)	O3—S2—N2	107.24 (11)
O2—S1—C1	107.05 (12)	O4—S2—C15	106.58 (12)
O1—S1—C1	107.14 (12)	O3—S2—C15	107.33 (12)
N1—S1—C1	107.67 (11)	N2—S2—C15	107.70 (11)
C13—N1—C7	119.10 (19)	C27—N2—C21	118.87 (19)
C13—N1—S1	117.41 (17)	C27—N2—S2	120.95 (16)
C7—N1—S1	119.11 (15)	C21—N2—S2	119.45 (15)
C2—C1—C6	119.8 (2)	C20—C15—C16	120.2 (3)
C2—C1—S1	120.3 (2)	C20—C15—S2	119.8 (2)
C6—C1—S1	120.0 (2)	C16—C15—S2	120.0 (2)
C3—C2—C1	119.5 (3)	C15—C16—C17	119.4 (3)
C3—C2—H2	120.3	C15—C16—H16	120.3
C1—C2—H2	120.3	C17—C16—H16	120.3
C4—C3—C2	120.3 (3)	C18—C17—C16	120.4 (3)
C4—C3—H3	119.8	C18—C17—H17	119.8
C2—C3—H3	119.8	C16—C17—H17	119.8
C5—C4—C3	120.2 (3)	C17—C18—C19	119.9 (3)
C5—C4—H4	119.9	C17—C18—H18	120.1
C3—C4—H4	119.9	C19—C18—H18	120.1
C4—C5—C6	120.7 (3)	C20—C19—C18	120.5 (3)
C4—C5—H5	119.7	C20—C19—H19	119.7
C6—C5—H5	119.7	C18—C19—H19	119.7
C5—C6—C1	119.5 (3)	C19—C20—C15	119.7 (3)
C5—C6—H6	120.2	C19—C20—H20	120.2
C1—C6—H6	120.2	C15—C20—H20	120.2
N1—C7—C8	110.77 (18)	N2—C21—C26	111.41 (18)
N1—C7—C12	114.24 (19)	N2—C21—C22	113.61 (19)
C8—C7—C12	110.8 (2)	C26—C21—C22	110.87 (19)
N1—C7—H7	106.9	N2—C21—H21	106.8
C8—C7—H7	106.9	C26—C21—H21	106.8
C12—C7—H7	106.9	C22—C21—H21	106.8
C7—C8—C9	111.2 (2)	C23—C22—C21	110.5 (2)
C7—C8—H8A	109.4	C23—C22—H22A	109.6
C9—C8—H8A	109.4	C21—C22—H22A	109.6
C7—C8—H8B	109.4	C23—C22—H22B	109.6
C9—C8—H8B	109.4	C21—C22—H22B	109.6
H8A—C8—H8B	108.0	H22A—C22—H22B	108.1
C10—C9—C8	111.7 (2)	C24—C23—C22	111.8 (2)
C10—C9—H9A	109.3	C24—C23—H23A	109.3
C8—C9—H9A	109.3	C22—C23—H23A	109.3
C10—C9—H9B	109.3	C24—C23—H23B	109.3
C8—C9—H9B	109.3	C22—C23—H23B	109.3
H9A—C9—H9B	107.9	H23A—C23—H23B	107.9
C11—C10—C9	111.5 (2)	C25—C24—C23	111.9 (2)
C11—C10—H10A	109.3	C25—C24—H24A	109.2
C9—C10—H10A	109.3	C23—C24—H24A	109.2
C11—C10—H10B	109.3	C25—C24—H24B	109.2
C9—C10—H10B	109.3	C23—C24—H24B	109.2
H10A—C10—H10B	108.0	H24A—C24—H24B	107.9
C10—C11—C12	111.8 (2)	C24—C25—C26	111.9 (2)
C10—C11—H11A	109.3	C24—C25—H25A	109.2
C12—C11—H11A	109.3	C26—C25—H25A	109.2
C10—C11—H11B	109.3	C24—C25—H25B	109.2
C12—C11—H11B	109.3	C26—C25—H25B	109.2
H11A—C11—H11B	107.9	H25A—C25—H25B	107.9
C11—C12—C7	110.5 (2)	C21—C26—C25	110.73 (19)
C11—C12—H12A	109.5	C21—C26—H26A	109.5
C7—C12—H12A	109.5	C25—C26—H26A	109.5
C11—C12—H12B	109.5	C21—C26—H26B	109.5
C7—C12—H12B	109.5	C25—C26—H26B	109.5
H12A—C12—H12B	108.1	H26A—C26—H26B	108.1
N1—C13—C14	113.5 (2)	N2—C27—C28	115.4 (2)
N1—C13—H13A	108.9	N2—C27—H27A	108.4
C14—C13—H13A	108.9	C28—C27—H27A	108.4
N1—C13—H13B	108.9	N2—C27—H27B	108.4
C14—C13—H13B	108.9	C28—C27—H27B	108.4
H13A—C13—H13B	107.7	H27A—C27—H27B	107.5
C13—C14—H14A	109.5	C27—C28—H28A	109.5
C13—C14—H14B	109.5	C27—C28—H28B	109.5
H14A—C14—H14B	109.5	H28A—C28—H28B	109.5
C13—C14—H14C	109.5	C27—C28—H28C	109.5
H14A—C14—H14C	109.5	H28A—C28—H28C	109.5
H14B—C14—H14C	109.5	H28B—C28—H28C	109.5
			
O2—S1—N1—C13	164.91 (17)	O4—S2—N2—C27	−151.80 (18)
O1—S1—N1—C13	35.1 (2)	O3—S2—N2—C27	−21.8 (2)
C1—S1—N1—C13	−79.84 (19)	C15—S2—N2—C27	93.4 (2)
O2—S1—N1—C7	−38.8 (2)	O4—S2—N2—C21	38.1 (2)
O1—S1—N1—C7	−168.55 (16)	O3—S2—N2—C21	168.11 (17)
C1—S1—N1—C7	76.49 (19)	C15—S2—N2—C21	−76.65 (19)
O2—S1—C1—C2	−148.1 (2)	O4—S2—C15—C20	144.5 (2)
O1—S1—C1—C2	−18.9 (2)	O3—S2—C15—C20	15.5 (2)
N1—S1—C1—C2	96.0 (2)	N2—S2—C15—C20	−99.7 (2)
O2—S1—C1—C6	32.3 (2)	O4—S2—C15—C16	−37.5 (2)
O1—S1—C1—C6	161.5 (2)	O3—S2—C15—C16	−166.5 (2)
N1—S1—C1—C6	−83.5 (2)	N2—S2—C15—C16	78.3 (2)
C6—C1—C2—C3	0.0 (4)	C20—C15—C16—C17	0.4 (4)
S1—C1—C2—C3	−179.6 (2)	S2—C15—C16—C17	−177.7 (2)
C1—C2—C3—C4	0.4 (4)	C15—C16—C17—C18	−1.1 (4)
C2—C3—C4—C5	0.0 (5)	C16—C17—C18—C19	1.0 (5)
C3—C4—C5—C6	−0.6 (5)	C17—C18—C19—C20	−0.2 (5)
C4—C5—C6—C1	0.9 (4)	C18—C19—C20—C15	−0.6 (5)
C2—C1—C6—C5	−0.6 (4)	C16—C15—C20—C19	0.5 (4)
S1—C1—C6—C5	179.0 (2)	S2—C15—C20—C19	178.5 (2)
C13—N1—C7—C8	−76.0 (3)	C27—N2—C21—C26	68.9 (3)
S1—N1—C7—C8	128.08 (19)	S2—N2—C21—C26	−120.80 (19)
C13—N1—C7—C12	49.9 (3)	C27—N2—C21—C22	−57.1 (3)
S1—N1—C7—C12	−106.0 (2)	S2—N2—C21—C22	113.1 (2)
N1—C7—C8—C9	−176.3 (2)	N2—C21—C22—C23	−176.43 (19)
C12—C7—C8—C9	55.9 (3)	C26—C21—C22—C23	57.2 (3)
C7—C8—C9—C10	−54.6 (3)	C21—C22—C23—C24	−55.4 (3)
C8—C9—C10—C11	53.9 (3)	C22—C23—C24—C25	53.6 (3)
C9—C10—C11—C12	−54.9 (3)	C23—C24—C25—C26	−53.3 (3)
C10—C11—C12—C7	56.1 (3)	N2—C21—C26—C25	175.45 (19)
N1—C7—C12—C11	177.6 (2)	C22—C21—C26—C25	−57.0 (3)
C8—C7—C12—C11	−56.5 (3)	C24—C25—C26—C21	55.0 (3)
C7—N1—C13—C14	107.4 (3)	C21—N2—C27—C28	93.3 (3)
S1—N1—C13—C14	−96.2 (3)	S2—N2—C27—C28	−76.8 (3)

## References

[bb1] Arshad, M. N., Zia-ur-Rehman, M. & Khan, I. U. (2009). *Acta Cryst.* E**65**, o2596. 10.1107/S1600536809038951 PMC2970422 21578031

[bb2] Berredjem, M., Régainia, Z., Djahoudi, A., Aouf, N. E., Dewinter, G. & Montero, J. L. (2000). *Phosphorus Sulfur Silicon Relat. Elem* **165**, 249–264.

[bb3] Bruker (2007). *APEX2* and *SAINT* Bruker AXS Inc., Madison, Wisconsin, USA.

[bb4] Lee, J. S. & Lee, C. H. (2002). *Bull. Korean Chem. Soc.* **23**, 167–169.

[bb5] Macrae, C. F., Edgington, P. R., McCabe, P., Pidcock, E., Shields, G. P., Taylor, R., Towler, M. & van de Streek, J. (2006). *J. Appl. Cryst.* **39**, 453–457.

[bb6] Sheldrick, G. M. (2008). *Acta Cryst.* A**64**, 112–122. 10.1107/S0108767307043930 18156677

[bb7] Soledade, M., Pedras, C. & Jha, M. (2006). *Bioorg. Med. Chem* **14**, 4958–4979. 10.1016/j.bmc.2006.03.014 16616505

[bb8] Spek, A. L. (2009). *Acta Cryst.* D**65**, 148–155. 10.1107/S090744490804362X PMC2631630 19171970

[bb9] Xiao, Z. & Timberlake, J. W. (2000). *J. Heterocycl. Chem* **37**, 773–777.

[bb10] Zia-ur-Rehman, M., Choudary, J. A., Elsegood, M. R. J., Siddiqui, H. L. & Khan, K. M. (2009). *Eur. J. Med. Chem* **44**, 1311–1316. 10.1016/j.ejmech.2008.08.002 18804313

